# Buffalo City Dispensary

**Published:** 1866-01

**Authors:** 


**Buffalo City Dispensary.**

*Officers for ensuing year*—President, Jason Sexton; Vice President, F. P. Wood; Treasurer, S. N. Callender; Secretary, Isaac D. White.

*Board of Managers*—Samuel N. Callender, Charles H. Coleman, C. W. Harvey, William Tweedy, George A. Moore, Isaac D. White, Jason Sexton, Jacob H. Koons, George Howard, George Jones, Samuel Cary, M. D., Francis P. Wood, James Brailey.

*Physicians*—2d ward, Dr. P. H. Strong; 3d ward, Dr. J. Boardman; 4th ward, Dr. J. Hauenstein; 6th ward, Dr. J. B. Samo; 7th ward, Dr. J. N. Brown; 8th ward, Dr. Sandford Eastman; 9th ward, Dr. C. C. Wyckoff; 10th ward, Dr. Thomas Lothrop; 11th ward, Dr. L. P. Dayton; 12th ward, Dr. Henry Nichell.

*Consulting Physicians*—Drs. Thomas F. Rochester and George N. Burwell.

*Consulting Surgeons*—Drs. Julius F. Miner and J. R. Lothrop.

*Apothecary*—Wm. H. Peabody, Clarendon Hotel block, corner Main and South Division streets.

